# Does Early Endoscopy Improve Mortality in Patients with Acute Non-variceal Gastrointestinal Bleeding? A Retrospective review

**DOI:** 10.7759/cureus.2246

**Published:** 2018-02-28

**Authors:** Umair Iqbal, Hafsa Anwar, Hunaiz Patel, Ahmad Chaudhary, Pascale Raymond

**Affiliations:** 1 Internal Medicine, Bassett Medical Center, Cooperstown, Ny; 2 Jinnah Sindh Medical University, Dow University of Health Sciences (DUHS), Karachi, Pakistan; 3 General Surgery, Bassett Medical Center, Cooperstown, Ny; 4 Gastroenterology, Bassett Medical Center, Cooperstown, Ny

**Keywords:** upper gastrointestinal bleeding, non-variceal gastrointestinal bleeding, urgent endoscopy, mortality

## Abstract

Introduction

Initial management of acute upper gastrointestinal bleeding (UGIB) aims towards aggressive fluid resuscitation to maintain hemodynamic stability. Existing evidence regarding the benefit of early endoscopy is unclear with some studies suggesting mortality benefits and some suggesting otherwise. The purpose of this study is to evaluate if there is any mortality benefit of doing early endoscopy within 24 hours of presentation.

Methods

From July 2013 to July 2016, 179 patients admitted with a diagnosis of non-variceal UGIB were retrospectively reviewed. Clinical variables including 30-day mortality were then compared between the patients who had endoscopy within 24 hours with those who had endoscopy after greater than 24 hours.

Results

Out of 179 patients admitted for non-variceal UGIB, 146 underwent endoscopy within 24 hours of presentation and 33 underwent endoscopy after 24 hours. The overall mortality associated with UGIB was 6.7% (12/179). There was no statistically significant difference found in 30-day mortality between the two groups (6.8% within 24 hours vs 6.1% after 24 hours). There was also no difference in 30-day readmission or rates of rebleeding among the two groups. The length of stay was also similar in both groups (6.0 days vs 6.1 days).

Conclusion

This study did not find any advantage of endoscopy within 24 hours on length of stay, rate of complications, and 30-day mortality. As hemostasis is achieved in almost 90% of patients with supportive management without any endoscopic intervention, focus should be made on aggressive fluid resuscitation to achieve hemodynamic stability before endoscopy.

## Introduction

Upper gastrointestinal bleeding (UGIB) is a common cause of hospitalization in the United States [1]. Inpatient mortality of UGIB has been reported to be as high as 14% [2]. Initial management of acute UGIB aims towards aggressive fluid resuscitation to maintain hemodynamic stability, proton pump inhibitors, blood transfusion, and anticoagulation reversal if needed [3]. Esophagogastroduodenoscopy (EGD) is a gold standard for the evaluation and management of the etiology of UGIB. Multiple guidelines recommend EGD within 24 hours of presentation of UGIB although existing evidence regarding the benefit of early endoscopy is controversial [4-7]. Multiple studies have shown urgent endoscopy in patients is not associated with improved mortality and outcomes [8-10]. Some studies revealed poor outcomes with early endoscopy in low-risk patients [11]. On the contrary, several studies revealed mortality benefit of urgent endoscopy and showed urgent EGD is associated with decreased length of stay and is associated with better outcomes [12-14].

A nationwide study on patients with UGIB showed early EGD to be associated with lower mortality, shorter length of stay, and lower healthcare cost [12]. Kumar et al. in a retrospective study in 361 patients showed a five-fold increased risk of inpatient mortality [11]. In a randomized controlled trial of 93 patients of UGIB there was no difference in mortality among urgent endoscopy (within six hours) and nonurgent endoscopy (within 48 hours), although there was increased detection of high-risk lesions in the urgent group [8]. The purpose of this study is to evaluate if there is any mortality benefit of doing urgent endoscopy within 24 hours of presentation in patients admitted with acute non-variceal UGIB.

## Materials and methods

We retrospectively reviewed electronic medical records of patients older than 18 years of age who were admitted to the Bassett Medical Center, Cooperstown, New York from July 2013 to July 2016. All patients over 18 years of age admitted with a diagnosis of acute non-variceal upper gastrointestinal bleeding (UGIB) were included in the study. Patients were identified by the International Classification of Diseases (ICD) 9 and ICD 10 codes. The study was reviewed by the institutional review board of the Bassett Medical Center and exempted for review due to the retrospective nature of the study.

Medical histories including history of coronary artery disease, previous congestive heart failure (CHF), diabetes, hypertension, and atrial fibrillation were recorded. Clinical variables including timing of endoscopy, length of stay, 30 days mortality from the day of admission, and 30 days readmission rates were abstracted from the chart review. Patients were analyzed in two groups: patients who underwent urgent endoscopy defined as endoscopic intervention within 24 hours of admission to the hospital and those who did not have endoscopic intervention within 24 hours (nonurgent). The primary outcome compared between the urgent and nonurgent endoscopy groups was mortality within 30 days. Secondary outcomes were hospital length of stay and 30 days readmission rates, which are considered as markers of poor healthcare quality.

Statistical analysis

To identify associations between urgent/nonurgent endoscopy and various patient and clinical characteristics, the Chi-square/Fisher’s exact test and independent samples t-tests were used. A p-value of < 0.05 was considered statistically significant. All analyses were carried out using Statistical Package for the Social Sciences (SPSS) Version 23.0 (IBM Corp., Armonk, NY).

## Results

Out of 179 patients admitted for non-variceal UGIB, 146 patients underwent endoscopy within 24 hours and 33 patients after 24 hours. The baseline and clinical characteristics of the patients are shown in Table 1. The mean age was similar between the two groups. There was a much higher percentage of males in the urgent endoscopy group. The mean hemoglobin in the entire cohort was 9.6 g/dl (9.6±2.9). Patients who underwent urgent endoscopy had lower hemoglobin on presentation compared to the nonurgent group, though it was not statistically significant (9.5 g/dl vs 10.4 g/dl, P=0.10). There was no difference in comorbidities including coronary artery disease, diabetes, chronic obstructive pulmonary disease (COPD), chronic kidney disease, and hypertension. There was also no statistical difference in the Charlson comorbidity score among the two groups (3.0 vs 2.7, P=0.95).

**Figure 1:**
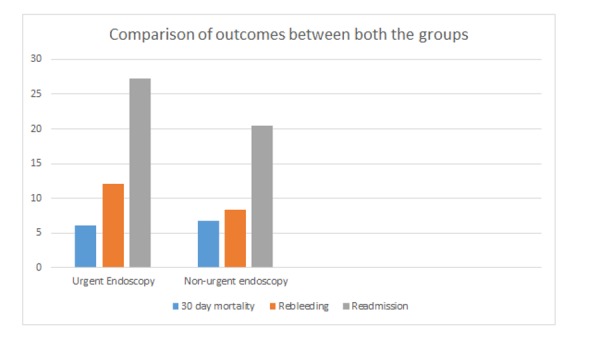
Comparison of primary and secondary outcomes between the two groups

The overall mortality associated with UGIB was 6.7% (12/179). There was no statistically significant difference found in 30-day mortality among the two groups (6.8% within 24 hours vs 6.1% after 24 hours, P=0.870). There was also no difference in 30-day readmission (20.5% in urgent endoscopy group and 27.3% in nonurgent endoscopy, P=0.39) and rates of rebleeding among the two groups (8.3% vs 12.1, P=0.48). The length of stay was also similar in both groups (6.0 days vs 6.1 days, P=0.18). Table 1 shows the univariate test results. Figure 1 compares the primary and secondary outcomes between the two groups.

**Table 1:** Results from univariate tests (chi-square/Fisher’s exact tests for categorical variables, t-tests for continuous variables)

Characteristics		Total (n=179)	Endoscopy				
			Urgent Endoscopy (n=33)		Nonurgent Endoscopy (n=146)		P-value
			n	%	n	%	
Age	Mean, SD	70±15	71.8		69.7		0.46
Sex	Male	72(40%)	18	54.5	54	37.0	0.063
BMI	Mean, SD	29.3±8.0	30.4		29.0		0.36
Hemoglobin on presentation	Mean, SD	9.6±2.9	10.4	3.2	9.5	2.8	0.10
LOS, days	Mean, SD	6.0±9.7	6.1		6.0		0.18
CSS	Mean, SD	2.9±2.6	2.7	2.9	3.0	2.5	0.95
Hypotension		26(14.45)	3	9.1	23	15.8	0.327
COPD		31(17.2%)	7	21.2	24	16.4	0.513
CAD		60(33.3%)	9	27.3	51	34.9	0.40
Diabetes		63(35%)	14	42.4	49	33.6	0.336
HTN		127(70.6%)	22	66.7	105	71.9	0.548
GERD		60(33.3%)	9	27.3	51	34.9	0.40
CKD		38(21.1%)	6	18.2	32	21.9	0.636
Atrial Fibrillation		51(28.3%)	8	24.2	43	29.5	0.549
CHF		43(23.9%)	8	24.2	35	24.0	0.97
Death in 30 days		12(6.67)	2	6.1	10	6.8	0.870
Death in 90 days		10(5.56)	1	3.0	9	6.2	0.47
Readmission		39(21.67)	9	27.3	30	20.5	0.39
Rebleeding		16(8.89)	4	12.1	12	8.3	0.48

## Discussion

UGIB is a common presentation and hospitalization rates are much higher than lower gastrointestinal bleeding [15]. The common causes of UGIB include gastric and duodenal ulcers, esophageal varices, portal gastropathy, and erosive esophagitis [16]. The timing of EGD in evaluation of UGIB is a topic of much controversy with some studies favoring urgent EGD while others did not show any improved outcomes. Our study did not show any significant short term mortality benefit in patients who underwent EGD within 24 hours of presentation. The findings of our study are in agreement with some prior studies that showed no mortality benefit with urgent endoscopy [8-11].

A retrospective study on 81 patients with UGIB secondary to peptic ulcer disease did not show any difference in outcomes including mortality, rates of rebleeding or length of stay but showed increased detection of high-risk bleeding lesions in the urgent endoscopy group [9]. Similar results were seen in another retrospective study on 189 patients, which compared outcomes between patients receiving endoscopy within eight hours versus those who received endoscopy between eight and 24 hours and revealed no difference in mortality or recurrent bleeding [10].

A retrospective, nationwide study including 1,789,532 patients who underwent early EGD had lower incidence of acute renal failure, hypovolemia, and acute respiratory failure [12]. Mortality was also lower in the early endoscopy group. Similar results were seen in another nationwide study that showed lower risk of mortality with early EGD [13]. A retrospective study on 909 inpatients revealed shorter length of stay and low rates of recurrent bleeding in patients who underwent early endoscopy [14].

In a nationwide cohort study on 12,601 patients, optimal timing of EGD was 12-36 in hemodynamically stable patients as mortality and timing of endoscopy showed a U-shaped association. Mortality trended higher outside a 12-36 hour period [17]. Hence optimal fluid resuscitation in patients before EGD improves outcomes in hemodynamically stable patients with UGIB. Kumar et al. in a retrospective study in 361 patients revealed no significant difference in mortality in high-risk patients with UGIB (Glasgow-Blatchford Bleeding Score (GBS) ≥12) who received urgent endoscopy. Interestingly, mortality trended higher in low-risk patients (GBS <12) who received urgent endoscopy [11]. These findings were contradictory to a study done by Lim et al. that revealed urgent EGD in high-risk patients (GBS≥12) is associated with low mortality [18].

Our study has several limitations and caution should be made in interpreting the results. First, the design of the study was retrospective. Our sample size was small compared to thousands of subjects included in prior studies. This may limit the interpretation of our results. We did not perform sub group analysis to compare difference in mortality between high-risk vs low-risk or hemodynamically stable vs hemodynamically unstable patients. This might impact the decision of timing of endoscopy as hemodynamically unstable high-risk patients might benefit from early endoscopic intervention.

## Conclusions

In patients with upper gastrointestinal bleeding, urgent endoscopy within 24 hours might not be associated with lower mortality, rate of rebleeding, 30 days readmission, and shorter length of stay. Efforts should be made to first achieve hemodynamic stability in these patients by optimal fluid resuscitation. Further randomized controlled trials are needed to clarify the association of urgent endoscopy in UGIB with mortality.

## References

[1] Tielleman T, Bujanda D, Cryer B (2015). Epidemiology and risk factors for upper gastrointestinal bleeding. Gastrointest Endosc Clin N Am.

[2] Colle I, Wilmer A, Le Moine O (2011). Upper gastrointestinal tract bleeding management: Belgian guidelines for adults and children. Acta gastro-enterologica Belgica.

[3] Kumar NL, Travis AC, Saltzman JR (2016). Initial management and timing of endoscopy in nonvariceal upper GI bleeding. Gastrointest Endosc.

[4] Hwang JH, Fisher DA, Ben-Menachem T (2012). The role of endoscopy in the management of acute non-variceal upper GI bleeding. Gastrointest Endosc.

[5] Laine L, Jensen DM (2012). Management of patients with ulcer bleeding. Am J Gastroenterol.

[6] Sung JJ, Chan FK, Chen M (2011). Asia-Pacific Working Group consensus on non-variceal upper gastrointestinal bleeding. Gut.

[7] Dworzynski K, Pollit V, Kelsey A, Higgins B, Palmer K (2012). Management of acute upper gastrointestinal bleeding: summary of NICE guidance. BMJ.

[8] Bjorkman DJ, Zaman A, Fennerty MB, Lieberman D, Disario JA, Guest-Warnick G (2004). Urgent vs. elective endoscopy for acute non-variceal upper-GI bleeding: an effectiveness study. Gastrointest Endosc.

[9] Schacher GM, Lesbros-Pantoflickova D, Ortner MA, Wasserfallen JB, Blum AL, Dorta G (2005). Is early endoscopy in the emergency room beneficial in patients with bleeding peptic ulcer? A "fortuitously controlled" study. Endoscopy.

[10] Tai CM, Huang SP, Wang HP (2007). High-risk ED patients with nonvariceal upper gastrointestinal hemorrhage undergoing emergency or urgent endoscopy: a retrospective analysis. Am J Emerg Med.

[11] Kumar NL, Cohen AJ, Nayor J, Claggett BL, Saltzman JR (2017). Timing of upper endoscopy influences outcomes in patients with acute nonvariceal upper GI bleeding. Gastrointest Endosc.

[12] Garg SK, Anugwom C, Campbell J (2017). Early esophagogastroduodenoscopy is associated with better Outcomes in upper gastrointestinal bleeding: a nationwide study. Endosc Int Open.

[13] Wysocki JD, Srivastav S, Winstead NS (2012). A nationwide analysis of risk factors for mortality and time to endoscopy in upper gastrointestinal haemorrhage. Aliment Pharmacol Ther.

[14] Cooper GS, Chak A, Way LE, Hammar PJ, Harper DL, Rosenthal GE (1999). Early endoscopy in upper gastrointestinal hemorrhage: associations with recurrent bleeding, surgery, and length of hospital stay. Gastrointest Endosc.

[15] Longstreth GF (1995). Epidemiology of hospitalization for acute upper gastrointestinal hemorrhage: a population-based study. Am J Gastroenterol.

[16] Enestvedt BK, Gralnek IM, Mattek N, Lieberman DA, Eisen G (2008). An evaluation of endoscopic indications and findings related to nonvariceal upper-GI hemorrhage in a large multicenter consortium. Gastrointest Endosc.

[17] Laursen SB, Leontiadis GI, Stanley AJ, Moller MH, Hansen JM, Schaffalitzky de Muckadell OB (2017). Relationship between timing of endoscopy and mortality in patients with peptic ulcer bleeding: a nationwide cohort study. Gastrointest Endosc.

[18] Lim LG, Ho KY, Chan YH (2011). Urgent endoscopy is associated with lower mortality in high-risk but not low-risk nonvariceal upper gastrointestinal bleeding. Endoscopy.

